# Impact of COVID-19 on mental disorder claims among healthcare workers

**DOI:** 10.1093/occmed/kqag002

**Published:** 2026-03-10

**Authors:** Joseph H Puyat, Mariah Allyson Banal, Daniel Vigo, Christopher B McLeod

**Affiliations:** School of Population and Public Health, Faculty of Medicine, University of British Columbia, V6T 1Z3, Canada; Centre for Advancing Health Outcomes, Providence Health Care, V6Z 1Y6, Canada; School of Population and Public Health, Faculty of Medicine, University of British Columbia, V6T 1Z3, Canada; School of Population and Public Health, Faculty of Medicine, University of British Columbia, V6T 1Z3, Canada; Centre for Advancing Health Outcomes, Providence Health Care, V6Z 1Y6, Canada; Department of Psychiatry, Faculty of Medicine, University of British Columbia, V6T 1Z3, Canada; School of Population and Public Health, Faculty of Medicine, University of British Columbia, V6T 1Z3, Canada

## Abstract

**Background:**

Healthcare workers (HCWs) face ongoing mental health challenges, which were aggravated during the COVID-19 pandemic due to increased workloads, exposure to trauma and heightened infection risks. Although self-reported data have indicated elevated mental health symptoms among HCWs, there is limited evidence based on official workers’ compensation claims to assess the true extent of work-related mental disorders in this group.

**Aims:**

To examine changes in workers’ compensation claims for mental disorders among HCWs and other workers before and throughout the first 2 years of the pandemic.

**Methods:**

Mental disorder compensation claims data from January 2019 to December 2021 among HCWs and other workers were obtained from the Workers’ Compensation Board of British Columbia. Monthly claim rates were calculated using denominator data derived from Statistics Canada’s Labour Force Survey. Changes in claim rates among HCWs were assessed through interrupted time series analysis using other workers as control and the onset of the pandemic as the event.

**Results:**

The analysis found no significant shifts in the incidence of mental disorder claims among HCWs during the pandemic. In contrast, a marginal increase in claim rates during the same period was observed in other workers.

**Conclusions:**

Unlike prior research based on self-reported data, this study found no evidence of an increase in mental disorder claims among HCWs during the pandemic. These results suggest that workers’ compensation claims data may not fully capture the broader mental health challenges experienced by HCWs, potentially due to underreporting or barriers to accessing claims.

## INTRODUCTION

Healthcare workers (HCWs) experience high levels of stress, burnout, anxiety and depression due to the demanding nature of their jobs, which is characterized by long work hours, heavy patient loads and emotionally intense environments [1]. The onset of the COVID-19 pandemic dramatically exacerbated these challenges due to increased workload, constant exposure to trauma and heightened infection risks [2,3]. Various studies using self-reported data have consistently documented worsening mental health among HCWs, with many noting sharp increases in symptoms of anxiety, depression and psychological distress [4,5]. However, it remains unclear how these mental health impacts translate into formal compensation claims.

Workers’ compensation data offer a valuable lens to assess severe or work-related mental disorders, yet few studies have examined mental disorder compensation claims (MDCs) during the COVID-19 pandemic, particularly among HCWs. The Canadian province of British Columbia (BC) provides a relevant context, as its compensation system requires a clinical diagnosis and adheres to specific legislative and policy criteria for a condition to be deemed compensable. In this study, we examined changes in accepted MDCs among HCWs and non-HCWs following the onset of the COVID-19 pandemic, hypothesizing a pronounced increase among HCWs.

## METHODS

We analysed cases of accepted MDCs using administrative data from the Worker’s Compensation Board of BC (WorkSafeBC), which manages workers’ compensation and covers 95% of the province’s workforce [6]. Monthly incident MDCs from 2019 to 2021 served as the numerator, while denominator data were drawn from Statistics Canada’s Labour Force Survey (LFS) [7]. MDCs were included in the analysis if they were clinically diagnosed (i.e. supported by a diagnosis from a psychiatrist or psychologist) and deemed work-related (i.e. predominantly caused by one or more significant workplace stressors rather than by typical employer decisions, e.g. termination) in accordance with workers’ compensation legislation and policy (see Online Supplement for details on how work-related mental health conditions are determined).

To assess the COVID-19 pandemic’s impact on MDCs, we performed a controlled interrupted time series analysis using a Poisson regression model [8] that included parameters for time (in months), a binary variable for pre/post pandemic onset (March 2020 [9]), an HCW/non-HCW indicator, and interaction terms to assess change in trends. Claims data were prepared in SAS v9.4 and LFS data in Stata/SE 17.0; interrupted time series analysis and plot generation were conducted in R version 4.4. Ethics approval was obtained from The University of British Columbia Behavioural Research Ethics Board (#H14-00847), and the study adhered to ethical guidelines for secondary data analysis, which does not require informed consent.

## RESULTS

Throughout 2019 to 2021, there were a total of 2171 MDC claims recorded in the WorkSafeBC data (see Table S1, available as Supplementary data at *Occupational Medicine* Online). In BC, females consistently comprised a higher proportion of HCWs (72–78%) compared with non-HCWs (44–52%). The median age of MDC claimants in BC (2017–2021) was lower for HCWs (42–47 years) than for non-HCWs (44–50 years). Before the COVID-19 pandemic, approximately 400 000 non-HCWs in BC were employed, on average, nearly double the average number of HCWs (approximately 200 000). By 2021, HCW employment in BC increased by 13%, while non-HCW employment declined slightly (Table S1, available as Supplementary data at *Occupational Medicine* Online).

**Figure 1.**
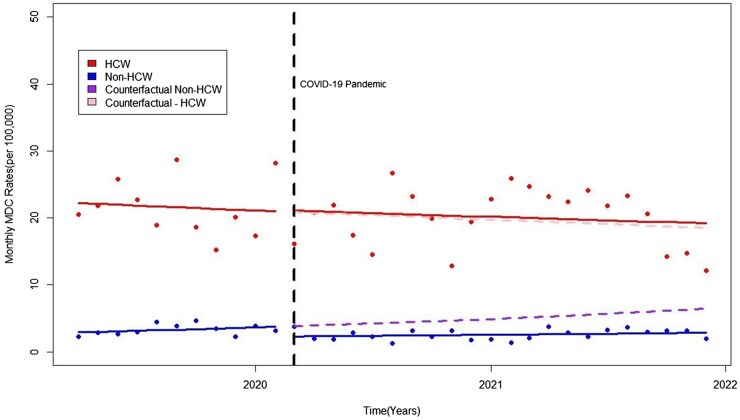
Mental disorder claims before and during the COVID-19 pandemic between HCWs and Non-HCWs. The counterfactual or dashed lines refer to the predicted values had the pandemic not occurred. Rates on the Y scale represent monthly mental disorder claims (MDC) per 100 000 workers.

Prior to the COVID-19 pandemic, MDCs among non-HCWs rose by about 2% (IRR = 1.02; 95% CI = 0.97–1.08; blue trend line in Figure 1) per month, while HCW MDCs declined by 1% per month (IRR = 0.99; 95% CI = 0.95–1.04, red trend line Figure 1). However, these trends were not significant, and the differences between them were minimal and not statistically significant (IRR = 0.97; 95% CI = 0.90–1.05).

At the onset of the COVID-19 pandemic (March 2020), there was a detectable drop in non-HCW MDC levels (IRR = 0.60; 95% CI = 0.37–0.98), whereas HCW levels were virtually unchanged (Table 1). MDC trends were stable, with non-HCW claims showing a non-statistically significant increase of about 1% monthly (IRR = 1.01; 95% CI = 0.99–1.03) and HCW claims showing a non-statistically significant slight decline (IRR = 0.99, 95% CI = 0.98–1.01).

**Table 1.** Model estimates from controlled interrupted time series analysis

Model estimates	Incidence rate ratio [95% confidence interval]
β0 (earliest pre-pandemic level, non-HCW)	2.90 [2.10–4.01]*
β1 (pre-pandemic slope, non-HCW)	1.02 [0.97–1.08]
β2 (level change, non-HCW)	0.60 [0.37–0.98]*
β3 (slope change, non-HCW)	0.99 [0.93–1.04]
β4 (earliest pre-pandemic level, HCW relative to non-HCW)	7.65 [5.07–11.54]*
β5 (pre-pandemic slope, HCW relative to non-HCW)	0.97 [0.90–1.05]
β6 (level change in HCW relative to non-HCW level change)	1.68 [0.89–3.20]
β7 (slope change in HCW relative to non-HCW slope change)	1.01 [0.94–1.10]
**Preintervention**	
Mean level in April 2019, non-HCWs	2.90 [2.10–4.01]*
Mean level in April 2019, HCWs	22.20 [17.42–28.29]*
Slope, non-HCWs	1.02 [0.97–1.08]
Slope, HCWs	0.99 [0.95–1.04]
**Postintervention**	
Mean level in March 2020, non-HCWs	2.3 [1.72–3.06]*
Mean level in March 2020, HCWs	21.08 [17.60–25.24]*
Slope, non-HCWs	1.01 [0.99–1.03]
Slope, HCWs	0.99 [0.98–1.01]

* *P* value < 0.05.

## DISCUSSION

This study found no significant changes in MDC levels or trends among HCWs during the COVID-19 pandemic. Among non-HCWs, MDC rates increased modestly pre-pandemic, but declined and then plateaued during the pandemic. These findings suggest that contrary to widely reported increases in mental health challenges among HCWs during the pandemic, work-related mental disorder claims remained stable.

A key strength of this study is its use of clinically diagnosed workers’ compensation data, offering an objective measure of the pandemic’s impact on workplace-attributed mental disorders. Including a non-HCW comparator further strengthens the interpretation, as this group worked in customer-facing essential occupations that required on-site presence and regular in-person contact throughout the pandemic (see Table S2, available as Supplementary data at *Occupational Medicine* Online). Study limitations include the use of broad occupational categories, a relatively short pre-pandemic period, and possible underrepresentation of HCWs’ mental health impacts in administrative data, since not all affected individuals sought care, received a diagnosis, or filed a claim. Additionally, claims involving both mental and physical injuries, or those combined with accepted COVID-19 claims, were excluded because it was not possible to determine whether the mental health outcome was secondary to the physical injury or to COVID-19 infection.

Comparing our findings to the broader literature is challenging because most studies rely on self-reported mental disorder symptoms rather than on clinically diagnosed disorders or accepted claims. One survey of Canadian nurses, for example, reported elevated rates of anxiety and depression early in the COVID-19 pandemic [10], in contrast to our findings. This discrepancy likely reflects the more restrictive nature of claims data, which require formal diagnosis and attribution to workplace exposures [10].

Several other factors may explain the absence of an increase in MDCs during the pandemic. First, expanded access to virtual psychological support [11,12] may have mitigated symptom severity or duration, reducing the need to file claims. Second, BC’s relatively lower COVID-19 burden early in the pandemic [13] and the prioritization of HCWs in the initial vaccine rollout may have buffered mental health impacts [14,15]. Third, labour dynamics may have influenced claim behaviour. A high number of personal illness or disability-related absences in 2020 suggests that some HCWs may have used other forms of leave or private benefits to manage mental disorder symptoms [13–16]. At the same time, HCW employment increased by 13% between 2020 and 2021 (see Table S1, available as Supplementary data at *Occupational Medicine* Online), possibly reflecting an influx of new workers entering the workplace to replace those that have left (18%). [17]

In conclusion, this study found no COVID-19 pandemic-related increase in MDCs among HCWs, contrasting with studies based on self-reported data. The findings highlight both the value and limitations of compensation claims data and the role of alternative mental health support. Future research should explore barriers to claim filing and long-term mental health outcomes to better support the mental health of HCWs during crises.

Key learning points **What is already known on this subject:**

- Previous studies have indicated that during the COVID-19 pandemic, healthcare workers, especially nurses and women, experience elevated levels of self-reported mental disorder symptoms.

**What this study adds:**

- Workers’ compensation claims data offer an objective and structured measure for tracking mental disorders among healthcare workers due to established policies and procedures.
- This study highlights differences between self-reported mental disorder symptoms and mental disorder claims data, providing a nuanced perspective on how mental health issues are documented.

**What impact this may have on practice or policy:**

- Future research should investigate barriers that healthcare workers face in filing mental disorder claims, particularly during crises like pandemics.
- Findings could inform policies designed to improve healthcare workers’ ability to seek support through compensation systems and mental health services.

## Supplementary Material

kqag002_Supplementary_Data

## Data Availability

Access to data provided by the Data Stewards is subject to approval, but can be requested for research projects through the Data Stewards or their designated service providers.
