# Supplementary material for: Impact of COVID-19 on mental disorder claims among healthcare workers
Source: Occup Med (Lond). 2026 Mar 10;76(2):150–3. doi: 10.1093/occmed/kqag002 (PMC13095214; doi:10.1093/occmed/kqag002)
Supplement: kqag002_Supplementary_Data [file kqag002_supplementary_data.docx]

# Additional Details about the Interrupted Time Series Analysis

To assess the COVID-19 pandemic’s impact on MDC incidence, we employed an interrupted time series analysis (ITSA) with a control group. This quasi-experimental design is commonly used to evaluate the long-term impacts of interventions or significant structural changes and events. (1) It is considered quasi-experimental because it allows for the evaluation of an intervention’s effect by comparing outcomes before and after the intervention without random assignment. It approximates a controlled experiment by using the pre-intervention trend as a baseline to detect changes attributable to the intervention.

Relative risks and their corresponding 95% confidence intervals (CI) were estimated using a Poisson regression model. This approach is consistent with methodologies employed in other studies analyzing injury counts.(2,3) No overdispersion was detected in the data, so no adjustments for this were made. The LFS denominator was included as an offset term in the Poisson model to generate incident rate ratio estimates based on incident MDCs per 100,000 workers. Offset terms are typically included to adjust for differences in population size when modeling count data, since the monthly MDCs will be dependent on population size. Autocorrelation and heteroscedasticity in the model were accounted for using the bootstrap technique with 2000 replications. Autocorrelation refers to the correlation of a variable with itself over time and could potentially violate one of the key assumptions of standard regression models regarding independence of observations. Heteroscedasticity is another potential violation of a key assumption in regression model and it pertains to the variance of residuals in a regression model not being constant across time.

The regression model for the ITSA, incorporating HCWs and non-HCWs as the two groups and the COVID-19 pandemic as the single interruption, is outlined as follows:

log(Yt) = β0 + β1Tt + β2Xt + β3XtTt + β4Z + β5ZTt + β6ZXt + β7ZXtTt + offset + εt (1)

In this equation, Yt represents the aggregated count of MDCs (outcome) at time (t); β0 denotes the intercept, representing the baseline MDC level among non-HCWs (control group); β1 indicates the pre-pandemic slope or trajectory of MDCs for non-HCWs; β2 captures the immediate change in level of MDCs among non-HCWs at the onset of the pandemic; β3 reflects the change in the slope of MDCs for non-HCWs during the pandemic period; β4 represents the difference in pre-pandemic baseline level of MDCs of HCWs compared to non-HCWs; β5 indicates the difference in the pre-pandemic slope of MDCs between HCWs and non-HCWs; β6 captures the difference in the level change of MDCs between HCWs and non-HCWs at the start of the pandemic; β7 represents the difference in the change in slope for HCWs and non-HCWs during the pandemic; εt represents the error term; Z indicates cohort assignment (HCW or non-HCW), ZT and ZX are interaction terms accounting for group differences over time and during the pandemic period, and X is a binary variable distinguishing the pre-pandemic and pandemic periods.

# Filing Compensation Claims in British Columbia (BC), Canada

When an injury occurs, the worker must give immediate notice and information to the employer as soon as practicable.(4) The information provided should contain specific details of the injury or disease on a form as supplied by WorkSafeBC. This information is important for WorkSafeBC to determine if compensation is applicable or not. The employer is also responsible for reporting the injury or death of a worker claimed to have arisen out of and in the course of employment.(4) A separate form provided by WorkSafeBC must be used by the employer to report the details of the injury or death of a worker. As per WorkSafeBC’s policy, there is a one-year limit to file a claim from the date of injury (Exceptions can be found in Section 152 of the BC Workers Compensation Act).

The Workers Compensation Act also describes what type of compensation is received by a worker. For short-term compensation, this refers to the worker’s average earnings. WorkSafeBC covers a certain percentage of this earnings. This type of compensation starts on the day of the injury and ends on the last day of the tenth week and is payable to the worker with a temporary disability resulting from work.(4) Should the injury last past the tenth week, the worker may be eligible for long-term compensation as deemed necessary by WorkSafeBC. If the worker did not have any type of earnings at the injury date, then WorkSafeBC will decide an appropriate manner to determine the compensation to the injured worker. Type of claims that receive this type of compensation is also known as time-loss claims because the injured worker is paid due to the time missed from work as a result of the injury.

# The Presumptive Clause in BC

The first presumption clause for mental disorders came into effect in May 2018 and policy changes to support these amendments came into effect in July 2018. At that time, the eligible occupations included firefighters, correctional officers, police officers, emergency medical assistants and sheriffs. These first responders often face traumatic events in the line of duty, including responding to dug overdose incidents. Coroners’ investigations have shown that most overdosed deaths occur in private residences (63%), with 13% in social or supportive housing and 9% among individuals experiencing homelessness. Frequent exposure to such distressing situations places first responders, particularly firefighters, paramedics, and police officers, at increased risk for developing post-traumatic stress disorder.

In April 2019, WorkSafeBC expanded the occupations to include nurses, healthcare aides, wildland firefighters and emergency response dispatchers. The inclusion of nurses appears to have resulted from the persistent advocacy of the British Columbia Nurses’ Union, which advocated for better mental health support for its members. Being included in the presumption clause means that workers in these occupations are not required to provide evidence linking their mental disorder to a specific workplace incident in order to access compensation.

The provision on assessing mental disorders as of the last amendment is as follows:

1. The mental disorder may be covered by either (a) if it is a reaction to one or traumatic events arising out of and in the course of employment or (b) if it is predominantly caused by significant work-related stressors arising out of and in the course of employment. The mental disorder cannot be caused by an employer’s decision relating to employment (e.g. performance evaluation, lay-offs, termination of employment).

2. Mental disorders must be diagnosed by a psychiatrist or psychologist

3. If the presumption clause applies to the injured worker, then it is presumed the mental disorder is caused by the employment unless the contrary is shown. In other words, the claim will be first considered under the presumptive clause.

In addition to meeting the criteria of the presumption clause, the injured worker and employer must report the claim in the same manner as any workplace injury. The claim is then assigned to a claims adjudicator who reviews the details and may involve the employer in the investigation process. If the claims adjudicator determines that the worker was exposed to a traumatic event or have a mental disorder predominantly caused by significant stressors described in 1, they will either arrange for a diagnostic assessment by a psychologist or psychiatrist or use an existing report as evidence. This assessment is required before WorkSafeBC can accept the claim. Once eligibility is confirmed, the adjudicator notifies the worker of the decision and outlines the next steps. Accepted claims may result in benefits such as wage loss compensation, health care treatment, or return to work support. WorkSafeBC applies the same laws and policies to all claims, regardless of the injury type.

Visual analysis of trends prior to the pandemic indicated that the implementation TPC contributed to a rise in MDCs between 2017 and 2019 (Figure S1 and S2). Hence, the introduction of the presumptive clause (TPC) is a potential confounder that need to be accounted for. To this end, the main results presented in the text was based on analysis that has pre-pandemic period beginning in April 2019 to minimize potential bias from the initial implementation of the policy. Demographic data were still provided for the entire 2017-2021 period (S1).

**References:**

1. Kontopantelis E, Doran T, Springate DA, Buchan I, Reeves D. Regression based quasi-experimental approach when randomisation is not an option: interrupted time series analysis. BMJ. 2015 Jun 9;350(jun09 5):h2750–h2750.

2. Fan JK, Amick BC, Richardson L, Scott-Marshall H, McLeod CB. Labor market and health trajectories during periods of economic recession and expansion in the United States, 1988‒2011. Scandinavian Journal of Work, Environment & Health. 2018;44(6):639–46.

3. Fan J, McLeod CB, Koehoorn M. Descriptive Epidemiology of Serious Work-Related Injuries in British Columbia, Canada. PLOS ONE. 2012 Jun 19;7(6):e38750.

4. Workers Compensation Act Chapter 1 Part 4 [Internet]. 2019 [cited 2023 Jun 9]. Available from: https://www.bclaws.gov.bc.ca/civix/document/id/complete/statreg/19001_04

**Figure S1. Sensitivity Analysis that Includes Data from 2017 to 2018 when the Presumptive Clause was Introduced**


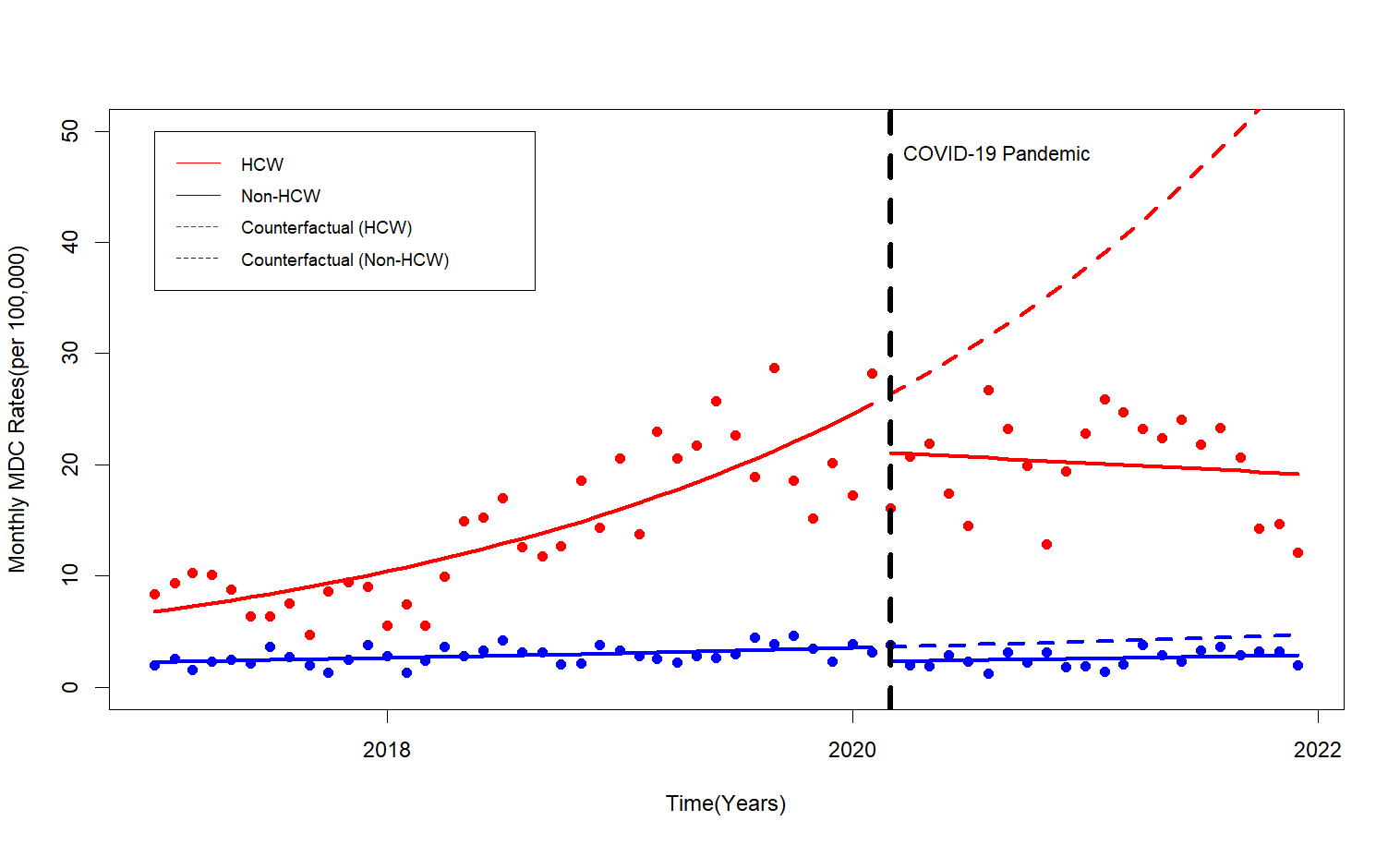


Figure S1 shows that including data from 2017 to 2018 when the presumptive clause was first introduced yields a biased estimate of the impact of the Covid-19 pandemic on mental disorder claims.

**Figure S2. Sensitivity Analysis 2 – Uncontrolled interrupted time series analysis for Healthcare workers with 2 intervention periods, one at the start of 2018 (presumptive clause) and one during 2020 (covid-19)**


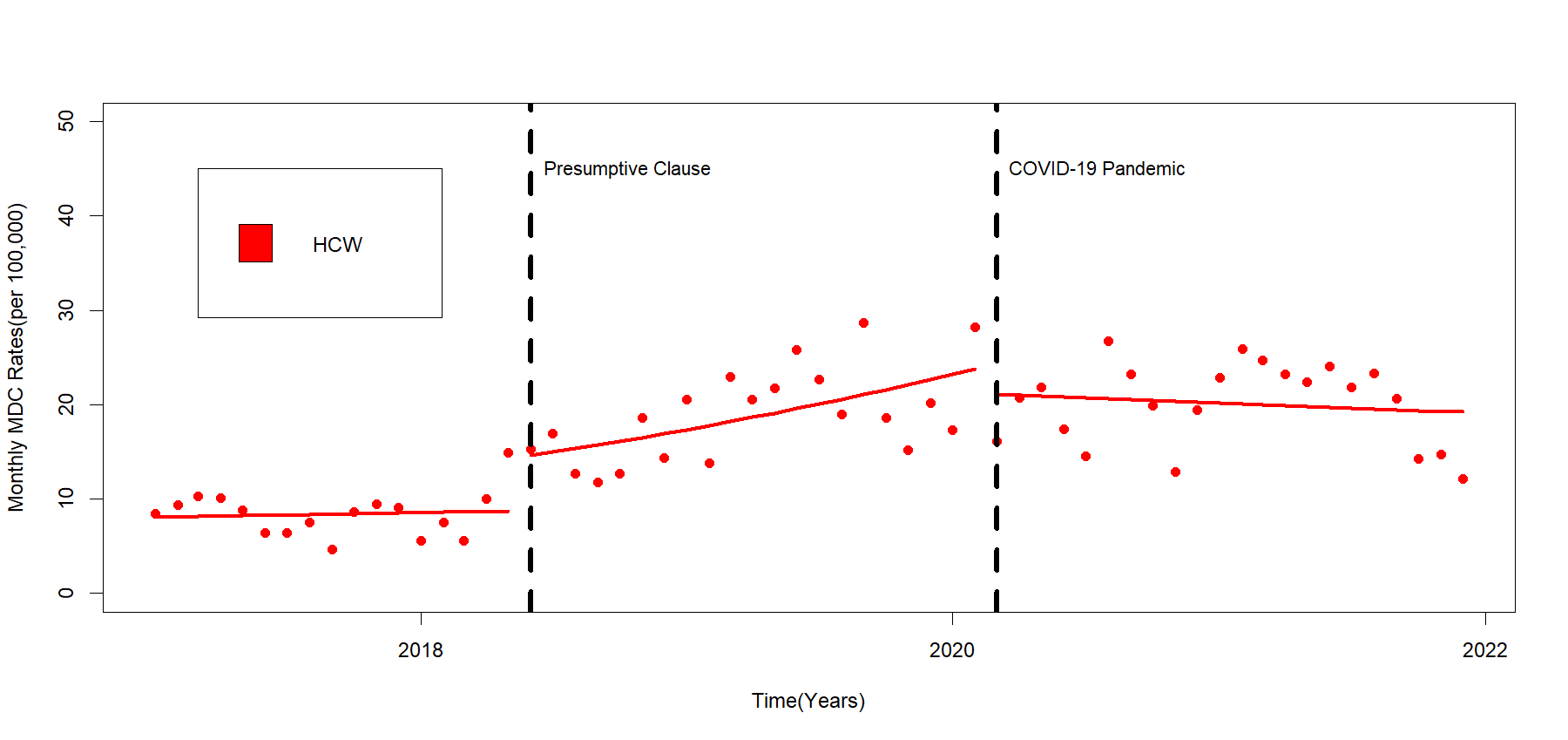


Figure S2 shows that the introduction of the presumptive clause in June 2018 has a significant impact on mental disorder claims and therefore serves as a confounder when examining the impact of the Covid-19 pandemic on mental disorder claims. Including data from 2019 only, as highlighted in the manuscript, will remove the confounding effect of the presumptive clause.

| **Table S1. Mental Disorder Claims and Employment Estimates by Year and Occupation Group** | | | | | | | |
| --- | --- | --- | --- | --- | --- | --- | --- |
| Year |  | Mental Disorder Claims | | |  | Employment Estimates | |
|  | Occupation | Total | Median Age | Females |  | Average | Females |
|  | Groups | (n) | (IQR) | % |  |  | % |
| 2017 | HCWs | 185 | 47(19) | 78 |  | 187,099 | 79 |
|  | Non-HCWs | 175 | 49(19) | 53 |  | 383,636 | 52 |
| 2018 | HCWs | 295 | 46(18) | 74 |  | 201,893 | 78 |
|  | Non-HCWs | 220 | 50(21.5) | 48 |  | 396,148 | 54 |
| 2019 | HCWs | 478 | 45(18) | 72 |  | 191,748 | 79 |
|  | Non-HCWs | 245 | 48(23) | 49 |  | 415,640 | 52 |
| 2020 | HCWs | 483 | 42(18) | 74 |  | 202,962 | 77 |
|  | Non-HCWs | 201 | 44(19) | 44 |  | 409,614 | 50 |
| 2021 | HCWs | 570 | 42(17) | 75 |  | 229,962 | 77 |
|  | Non-HCWs | 194 | 46(20) | 46 |  | 407,799 | 50 |
| MDC was derived from WorkSafeBC Claims Data. Employment Estimates was derived from the Labour Force Survey. | | | | | | | |

| **Table S2. NOC 2011 Code for Healthcare Workers and Non-healthcare Workers** | |
| --- | --- |
| **Healthcare workers** | **Description** |
| 30 | Professional occupations in nursing |
| 31 | Professional occupations in health (except nursing)^a^ |
| 32 | Technical occupations in health^b^ |
| 34 | Assisting occupations in support of health services^c^ |
| **Non-healthcare workers** |  |
| 64 | Sales representatives and salespersons - wholesale and retail trade |
| 65 | Service representatives and other customer and personal services occupations­^d^ |
| 66 | Sales support occupations^e^ |
| 67 | Service support and other service occupations^f^ |
| 75 | Transport and heavy equipment operation and related maintenance occupations |
| ^a This includes physicians, dentists, chiropractors and other health and diagnosing professionals^  ^b This includes medical technologists, technical occupations in dental, licensed practical nurses, massage therapists^  ^c This includes dental assistants, nurse aids, and orderlies^  ^d This includes occupations in food, beverage, tourism, customer and information services.^  ^e This includes cashiers, service station attendance, store shelf stockers, store clerks and order fillers.^  ^f This includes food counter attendants, kitchen helpers, and cleaners.^ | |

| Table S3. Mental Disorder Claims by Occupation Group 2017-2021 | |
| --- | --- |
| **Occupation Group** | **Mental Disorder Claims**  **n (%)** |
| *Healthcare Workers* |  |
| Professional occupations in nursing | 785 (39%) |
| Professional occupations in health | 23 (1%) |
| Technical Occupations in health | 874 (43%) |
| Assisting occupations in support of health services | 329 (16%) |
| *Non-healthcare Workers* |  |
| Sales representatives and salespersons - wholesale and retail trade | 93 (9%) |
| Service representatives and other customer and personal services occupation | 217 (21%) |
| Sales support occupations | 69 (7%) |
| Service support and other service occupations | 147 (14%) |
| Transport and heavy equipment operation and related maintenance occupations | 509 (49%) |

| **Table S4. List of Mental Disorder Diagnoses by ICD-9 Code** | |
| --- | --- |
| **ICD-9 Code** | **Diagnosis** |
| 300 | Anxiety disorder, unspecified |
| 300.01 | Panic disorder |
| 300.02 | Generalized Anxiety Disorder |
| 300.09 | Other mixed anxiety disorders |
| 300.09 | Other specified anxiety disorders |
| 296.31 | Major depressive disorder, recurrent, mild |
| 296.32 | Major depressive disorder, recurrent, moderate |
| 296.33 | Major depressive disorder, recurrent severe without psychotic features |
| 296.34 | Major depressive disorder, recurrent, severe with psychotic features |
| 296.35 | Major depressive disorder, recurrent, in remission |
| 296.3 | Other recurrent depressive disorders |
| 296.35 | Major depressive disorder, recurrent, unspecified |
| 309.81 | Post-traumatic stress disorder |
| 309 | Adjustment disorders |
| 309.02 | Adjustment reaction |
| 308.9 | Reaction to Severe Stress |
| 309.81 | Post-traumatic stress disorder, chronic |
| 311 | Depression, unspecified |
|  | |
